# Biofilm Interaction Mapping and Analysis (BIMA) of Interspecific Interactions in *Pseudomonas* Co-culture Biofilms

**DOI:** 10.3389/fmicb.2021.757856

**Published:** 2021-12-09

**Authors:** Suzanne M. Kosina, Peter Rademacher, Kelly M. Wetmore, Markus de Raad, Marcin Zemla, Grant M. Zane, Jennifer J. Zulovich, Romy Chakraborty, Benjamin P. Bowen, Judy D. Wall, Manfred Auer, Adam P. Arkin, Adam M. Deutschbauer, Trent R. Northen

**Affiliations:** ^1^Environmental Genomics and Systems Biology, Lawrence Berkeley National Laboratory, Berkeley, CA, United States; ^2^Department of Biochemistry, University of Missouri, Columbia, MO, United States; ^3^Lawrence Berkeley National Laboratory, Joint Genome Institute, Berkeley, CA, United States

**Keywords:** biofilm, mass spectrometry, mutant fitness profiling, consortia, morphology, microbial interaction, metabolomics, exometabolomics

## Abstract

Pseudomonas species are ubiquitous in nature and include numerous medically, agriculturally and technologically beneficial strains of which the interspecific interactions are of great interest for biotechnologies. Specifically, co-cultures containing *Pseudomonas stutzeri* have been used for bioremediation, biocontrol, aquaculture management and wastewater denitrification. Furthermore, the use of *P. stutzeri* biofilms, in combination with consortia-based approaches, may offer advantages for these processes. Understanding the interspecific interaction within biofilm co-cultures or consortia provides a means for improvement of current technologies. However, the investigation of biofilm-based consortia has been limited. We present an adaptable and scalable method for the analysis of macroscopic interactions (colony morphology, inhibition, and invasion) between colony-forming bacterial strains using an automated printing method followed by analysis of the genes and metabolites involved in the interactions. Using Biofilm Interaction Mapping and Analysis (BIMA), these interactions were investigated between *P. stutzeri* strain RCH2, a denitrifier isolated from chromium (VI) contaminated soil, and 13 other species of pseudomonas isolated from non-contaminated soil. One interaction partner, Pseudomonas fluorescens N1B4 was selected for mutant fitness profiling of a DNA-barcoded mutant library; with this approach four genes of importance were identified and the effects on interactions were evaluated with deletion mutants and mass spectrometry based metabolomics.

## Introduction

Consortia based systems in biotechnologies are widespread, however, controlling them is challenging due to the genomic and metabolomic complexities of the interactions. Characterization of the genes and metabolites involved in the interactions opens up the possibility for improved consortia functionality by use of engineered strains and culture condition metabolite amendments. Previously, metabolomics of adjacently printed cultures of *P. stutzeri* and *Shewanella oneidensis* were analyzed using replication-exchange-transfer and nanostructure initiator mass spectrometry; however, this approach does not elucidate the genes important for the interactions (Louie et al., 2013). The ability to incorporate genomics into these types of approaches will allow for a better understanding of the interactions involved. Barcoded mutant libraries are proving to be a powerful tool for the discovery of the genetic determinants of co-culture fitness (Goodman et al., 2011; Kosina et al., 2016). Next-generation sequencing enables rapid profiling of the abundance of barcodes mapped to specific genes in transposon mutant libraries under a wide range of environmental conditions (Wetmore et al., 2015; Price et al., 2018) and when integrated with metabolomics, provides rapid functional assignment of transport and metabolic processes (Baran et al., 2009) important in microbial interactions.

Pseudomonas are a diverse genus of microbes that have been isolated from all over the world of which both beneficial and pathogenic strains have been identified (Peix et al., 2009; Silby et al., 2011). As common soil-dwelling microorganisms, they are important constituents of microbial ecosystems and rhizosphere environments (Garbeva et al., 2004; Sørensen and Nybroe, 2004; Moore et al., 2006; Li et al., 2013). *Pseudomonas stutzeri*, a model denitrifying pseudomonas, can grow in diverse conditions (Lalucat et al., 2006) and its use has been demonstrated in a number of bioremediation processes, including phenol (Jiang et al., 2014), carbon tetrachloride (Sepúlveda-Torres et al., 2002), uranium (Han et al., 2012), polycyclic aromatic hydrocarbons (Moscoso et al., 2012a), and diesel oil (Kaczorek et al., 2012). Additionally, *P. stutzeri* has been identified in and used in a number of consortia-based applications, such as bioremediation (Nguyen et al., 2008; Brodie et al., 2011; Gałazka et al., 2012), wastewater denitrification (Clarens et al., 1998; Liu et al., 2006), aquaculture water quality management (Erna et al., 2013; Deng et al., 2014), as a plant growth promoter for the biocontrol of phytopathogens (Cottyn et al., 2009; Shen et al., 2013), and manufacturing/municipal waste management (Miyahara et al., 2012; Moscoso et al., 2012b). The use of *P. stutzeri* biofilms for various bioremediation efforts has potential benefits in terms of activity and yields for copper removal (Badar et al., 2013), drinking water denitrification (Lazarova et al., 1992) and naphthalene (Zhao et al., 2016), and phenol (Viggiani et al., 2006) degradation. Given this widespread utilization in both microbial consortia systems and biofilms, *P. stutzeri* was selected to demonstrate the deconstruction of its interactions with other pseudomonas in biofilm-based co-cultures into the genetic and chemical aspects influencing the co-colony fitness.

**FIGURE 1:**
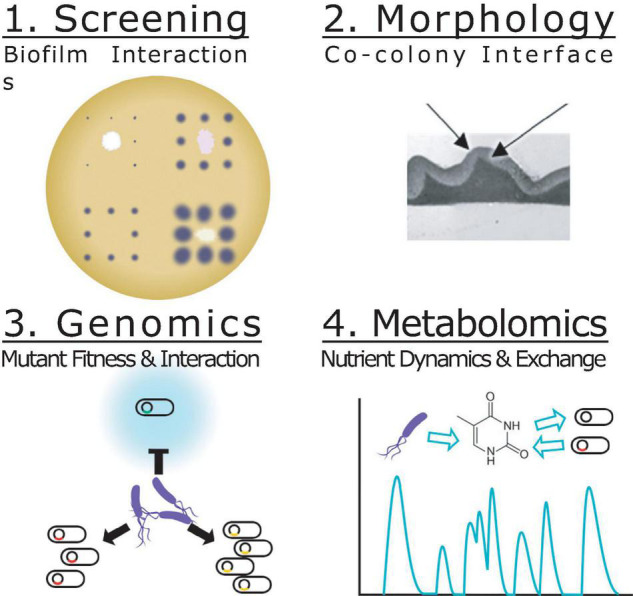
Biofilm Interaction Mapping and Analysis (BIMA) of the biochemical and biophysical interactions required for co-colony fitness. Screening (step 1): An acoustic printer or automated liquid handling system is used to print underlay colonies of different strains spaced apart to reduce interactions and a single strain grid of overlay colonies to evaluate the macroscopic interactions of the co-colony formation including: inhibition of colonization, direct overlay growth, morphology, color and motility. Morphology (step 2): Transmission electron microscopy is used to evaluate the interface between the two co-colony strains and the changes over time. Genomics (step 3): Mutant fitness profiling of a DNA-barcoded transposon mutant library of the overlay colony allows for investigation of genes important for co-colony formation and fitness. Metabolomics (step 4): Mass spectrometry based metabolomics is used to analyze the metabolites consumed/released in the co-culture vs. mono-culture.

Here we introduce Biofilm Interaction Mapping and Analysis (BIMA), an integrated platform of automated colony printing, barcoded mutant library profiling and metabolomics to discover and deconstruct the interactions within biofilm-based consortia (Figure 1). Overlaid colonies were printed using an automated liquid handling system to investigate the morphological, inhibitory and invasive interactions between *P. stutzeri* and other pseudomonas soil isolates in a lab model for a biofilm-based consortium. The overlaid colonies were further analyzed using transmission electron microscopy to investigate changes in ultrastructural organization at the species interface over time. Using a DNA-barcoded mutant transposon library of *P. stuzeri* strain RCH2, we identified genes associated with the fitness of the RCH2 in the co-colony. Additionally, exometabolomic analysis was used to evaluate the exchange of metabolites between RCH2 and another pseudomonas strain. We foresee these tools being valuable resources for both the understanding of natural interactions between pseudomonas in microbial communities and in the development of biofilm-based biotechnological applications.

## Materials and Methods

### Bacterial Strains and Growth Conditions

*Pseudomonas stutzeri* RCH2 was isolated from chromium(VI) contaminated groundwater at the Department of Energy Hanford 100 Area, Benton County, WA (Han et al., 2010). Thirteen additional pseudomonas strains were isolated from Oak Ridge National Laboratory, Field Research Center, TN under conditions indicated in Supplementary Table 1. Liquid cultures were inoculated into 3-(N-morpholino)propanesulfonic acid (MOPS) buffered casein yeast magnesium broth (Mb-CYM): 10 g/L pancreatic digest of casein, 5 g/L Difco yeast extract, 1 g/L MgSO_4_ heptahydrate, 10 mM MOPS. Mb-CYM agar was prepared with the addition of 1.5% w/v of agar. Unless otherwise noted, culture conditions were as follows. Strains were maintained as glycerol stocks; revived cultures were plated onto agar plates, incubated at 30°C for 24 h and stored at 4°C. Starter cultures for experiments were prepared from single colonies inoculated into liquid Mb-CYM broth and cultured aerobically with shaking at 30°C overnight. Uninoculated and unstreaked but incubated cultures/plates were used as negative controls for contamination. In figures and text, *Pseudomonas stutzeri* RCH2 is referred to as culture #1, while the 13 other strains are referred to as cultures #2–14, and uninoculated controls as culture #15 as indicated in Supplementary Table 1.

### Printed Colony Biofilm Morphology Screening Assay

**FIGURE 2:**
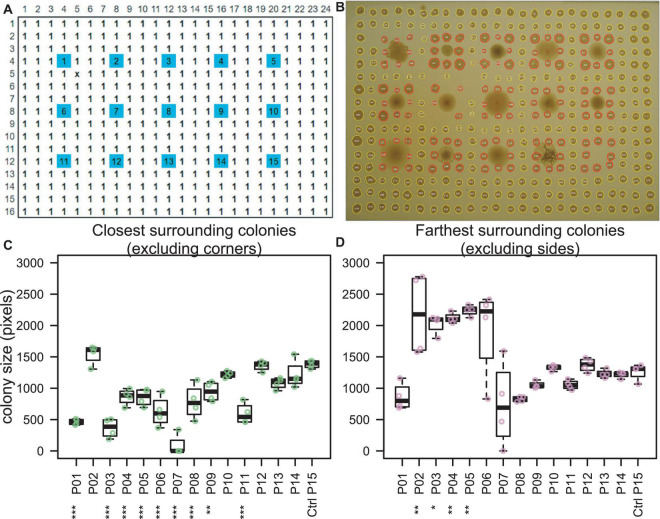
Colony interaction screening. **(A)** Fourteen Pseudomonas “effector” strains (spots #1–14) and one uninoculated control medium (spot #15) were printed from liquid cultures onto an agar plate (blue). After 2 days of incubation, the interaction strain, *P. stutzeri* RCH2 (#1) was printed in all other positions (white). **(B)** Image taken at 6 days total incubation time indicated inhibited growth of *P. stutzeri* RCH2 around strains 1 (self), 3, 4, 5, 6, 7, 8, 9, and 11. **(C,D)** *P. stutzeri* RCH2, the interaction strain, colony size was used to evaluate which effector strains had a significant (ANOVA, Dunnett’s test, ***p < 0.001, **p < 0.01, *p < 0.05) effect on the growth of the neighboring RCH2 colonies. One effector strain (#2), N1B4, with the largest mean surrounding colony area and which did not inhibit RCH2, was selected for further morphological and metabolic interaction with RCH2. When comparing all surrounding colonies, only #7 was significantly different from control (Supplementary Figure 2).

Rectangular petri dishes with ANSI standard dimensions were poured with agar to a height of 5 mm. Overnight cultures of the 14 effector strains (including RCH2) and uninoculated control broth (Supplementary Table 1) were diluted 1:50 in fresh Mb-MYM and then added to a 96-well plate. The plates were loaded onto a Hamilton Vantage Liquid Handling System equipped with a 96 pipetting head which was used to dip pipette tips into the liquid cultures and press/print them against the surface of the agar in a grid; a single colony of each unique strain was printed in a 3 × 5 grid (Figure 2A). The lidded-plate was sealed with Parafilm and incubated at 30°C for 2 days once visible colonies had formed of similar size (Supplementary Figure 1). Printing was repeated using only *P. stutzeri* RCH2 (the interaction strain) in all well positions of a 16 × 24 grid (ANSI standard dimensions of a 384 well plate), excluding the positions from the original print at time 0 (Figure 2A). Cultures were incubated for an additional 4 days prior to imaging using a digital camera. Image analysis was performed using ImageJ (Schneider et al., 2012). Briefly, the image was converted to 16-bit grayscale, corrected for uneven lighting using the FFT bandpass filter (filter large structures to 70 pixels and small structures to 5 pixels, 5% tolerance, autoscale and saturate after filtering), auto thresholded to black and white, and then the colonies were measured using the Analyze Particles tool (colonies missed by the automated selection were manually outlined and then measured). Measurements included area and position. Colony areas of surrounding (8 colonies in a square around the center interaction colony) and closest/farthest neighbors (sides/corners of the square) were compared with the colonies surrounding the control uninoculated position (#15) using Dunnett’s multiple comparison procedure using R version 3.6.2 (Hothorn et al., 2008; R Core Team, 2014).

### Co-colony Overlay Morphology With Light Microscopy and Transmission Electron Microscopy

Overnight culture of *Pseudomonas* sp. FW300-N1B4 (N1B4) was manually pipetted (10 μL) onto an agar plate and incubated at 30°C overnight. Overnight culture of *P. stutzeri* RCH2 was manually pipetted (10 μL) over the top of the N1B4 colony with care to not puncture the colony surface and to avoid allowing the droplet to spill over the sides. For time course analysis of macroscopic morphology, the rim of the petri dish was sealed with parafilm and placed on a black velvet cloth under a Leica M165FC microscope with a Planapo 1.0× objective lens. Images were acquired every 2 min for a 24 h period with a Leica DFC400 1.4 megapixel ccd sensor digital camera set at a 9 mm frame width. For microscopic colony interface analysis, after overnight growth of the overlay, the colony and underlying agar was cut out from the plate and fixed for 2 h with 2.5% glutaraldehyde in 0.1 M sodium cacodylate buffer (pH 7.2). Fixed samples were stained with 5 mM ruthenium red in 0.1 M sodium cacodylate buffer (pH 7.2) for 1 h and post-fixed with 1% osmium tetroxide for 1 h. Following staining, samples were dehydrated through a graded ethanol series (20, 40, 60, 80, 90, 100, 100, and 100%) followed by infiltration with an Embed-812 epoxy resin (Electron Microscopy Sciences):acetone series of 1:3 for 2 h, 2:3 for 2 h, and 100% resin overnight. Samples were then heat polymerized in 100% resin with N,N-dimethylbenzylamine accelerant for 2 h at 85°C. 100 nm sections were cut and sectioned using a Leica UC6 Ultra Microtome (Leica Microsystems Inc., Buffalo Grove, IL, United States) and then stained sequentially with 2% methanolic uranyl acetate and Reynolds’ lead citrate for 5 min each. Images were collected using a FEI Tecnai 12 transmission electron microscope (FEI Company, Hillsboro, OR, United States).

### Fitness Profiling of RCH2 Mutants in the Presence of Other Pseudomonads

The construction of the *P. stutzeri* RCH2 DNA-barcoded transposon mutant was previously described (Wetmore et al., 2015). Overnight culture of *Pseudomonas* sp. FW300-N1B4 was manually pipetted (10 μL) in triplicate onto LB agar plates. The plates were incubated overnight and then 5 μL of the *P. stutzeri* RCH2 barcoded transposon mutant library (in LB media) starter culture at an OD_600_ of 0.9 was spotted as overlay colonies on top of the three replicates of the underlay colonies. An aliquot of the starter culture was used for the initial RCH2 mutant abundances. Colonies were then carefully scraped and frozen for storage and subsequent analysis of the final *P. stutzeri* RCH2 mutant abundances. Gene fitness scores were calculated by comparing the initial and final mutant abundances as determined by deep sequencing of the DNA barcodes, as previously described (Wetmore et al., 2015).

### Mutant Construction

The four mutants from the pooled fitness assay with the largest absolute differential fitness where fitness of RCH2 with N1B4 was less than fitness of RCH2 on agar alone were selected for constructing gene deletion mutant strains. Deletion mutants (Supplementary Table 2) for gamma-glutamyl phosphate reductase, OHCU decarboxylase, formyltetrahydrofolate deformylase, and glutamate 5-kinase were constructed by conjugation of unstable, marker-exchange plasmids into *P. stutzeri* RCH2, as previously described (Vaccaro et al., 2016) with modifications as follows. All plasmids and primers (IDT, Newark, NJ) used are listed in Supplementary Tables 3, 4, respectively. Briefly, deletion cassettes, containing kanamycin resistance gene (npt) flanked by chromosomal regions up and downstream of the gene to be deleted, were assembled using the “gene SOEing” technique (Horton et al., 1990). Cloned homologous regions were sequenced at the DNA core facility at the University of Missouri, Columbia, and compared with the published sequence for *P. stutzeri* RCH2 (Chakraborty et al., 2017). Cassettes and the template plasmid pM07704 were amplified by polymerase chain reaction (PCR) with Herculase II DNA polymerase (Stratagene). Marker-exchange plasmids were generated by ligation of the PCR products in α-select cells (Bioline) using the SLIC cloning method (Li and Elledge, 2007). The plasmids were isolated and transformed into *E. coli* strain WM3064, and then transferred to RCH2 via conjugation (Fels et al., 2013). The plasmids, containing up/downstream chromosomal regions flanking npt, allow for exchange of npt with the gene of interest in RCH2 via double homologous recombination. Exconjugates were selected on 50 μg/mL kanamycin solid medium, then screened for spectinomycin (100 μg/mL) sensitivity to ensure no single recombination isolates were selected. Deletion strains were confirmed by Southern blot analysis. One isolate for each deletion of interest was retained while the other isolates were discarded. All strains were frozen as early stationary phase cultures in 10% (v/v) glycerol. RCH2 mutants used for experiments were maintained in Mb-CYM broth or Mb-CYM agar with 50 μg/mL kanamycin sulfate; kanamycin was not included in subsequent cultures for metabolomics analysis.

### Mutant Co-colony Morphology Analysis

Overnight cultures of RCH2 (wild-type and mutants) and N1B4 (500 μL) were centrifuged (5,000 × g × 3 min at RT) to collect cells, then washed 2 times in DPBS (resuspension in 500uL DPBS, centrifugation at 5,000 × g × 3 min at RT) with final resuspension adjusted to an OD (600 nm, 1 cm) of 0.5. Uninoculated control medium was washed and resuspended in a similar manner to account for carryover of nutrients from tube surfaces and residual volumes. Cultures (“underlays”) were manually spotted (2 μL) onto Mb-CYM agar plates in a 7 × 7 format and incubated overnight at 30°C. Fresh cultures were inoculated from agar stock plates into Mb-CYM. Overlay colonies were diluted and washed in the same manner as for the underlays and then carefully spotted on top of the underlay colonies. Images were taken using a digital camera of whole plates and through a LEICA M165 FC stereo microscope (2x objective) at 24 h (0 h of overlays), 48 h (24 h of overlays), 72 h (48 h of overlays). Morphological differences were visually evaluated.

### Metabolomics Analysis

Wild-type and mutant strains of *P. stutzeri* RCH2 were cultured in “spent” N1B4 medium (sterile filtered N1B4 culture supernatant) in liquid culture and then exometabolites were collected by extraction in methanol (Want et al., 2006; Baran et al., 2013; Sitnikov et al., 2016) for LCMS analysis. For collection of “spent” medium, overnight liquid cultures of RCH2 wild-type, RCH2 mutants, and N1B4 wild-type and uninoculated control medium were centrifuged (3,000 × g× 10 min) to pellet cells. Supernatants were sterile filtered (0.22 μm), supplemented with 1x Wolfe’s vitamins and minerals (ATCC) and stored at 4°C overnight. Additional overnight cultures were diluted, washed and adjusted as described above for mutant co-colony morphology analysis, except the final OD was adjusted to 0.12 in DPBS, 20 μL of which was added to 100 μL of spent medium in 96 well plates. Cultures were prepared in triplicates for all combinations of mutants and wild-type RCH2 on spent N1B4 and fresh Mb-CYM and for N1B4 on all combinations of spent mutants and wild-type RCH2 and fresh Mb-CYM; uninoculated controls were included for each spent medium and positive growth controls were included for each strain on fresh (non-spent) medium. Cultures were incubated for 20 h in a BioTek plate reader with OD readings taken every 30 min at 600 nm. Cross-feeding medium from the culture was then collected via centrifugation (3,000 × g for 5 min), transferred to a new plate that was sealed with heated foil (4titude), and frozen at −80°C. Holes were pierced in the foil with an 18 gauge needle and then supernatants were lyophilized to dryness. Dried material was resuspended in 200 uL of internal standard mix (15 μM 13C, 15N amino acid mix, 10 μg/mL 13C-mannitol, 13C-trehalose and 2 μM 15N4-inosine, 15N5-adenine, 13C4-15N2-uracil, 15N4-hypoxanthine, 13C4-15N2-thymine) in LCMS grade methanol, resealed, vortexed, sonicated in a room temperature water bath for 10 min, rechilled at −80°C for 5 min, and centrifuged 3,000 × g for 5 min to pellet insoluble material. Supernatants were filtered (0.22 μm, PVDF) using an Apricot positive pressure filtration device. Filtrates were then arrayed into 50 μL aliquots in a 384 well plate for LCMS analysis.

### LCMS Analysis of Exometabolites From Spent Media Fed Cultures

Extracts of polar metabolites were analyzed using hydrophilic interaction chromatography—mass spectrometry. Metabolites were retained and separated on an InfinityLab Poroshell 120 HILIC-Z column (Agilent, 683775-924, 2.7 μm, 150 × 2.1 mm) using an Agilent 1290 UHPLC. Samples, held at 4°C, were injected at 4 μL each; the column temperature was held at 40°C and flow rate was held at a constant 0.45 mL/min. Following injection, a gradient of mobile phase A (5 mM ammonium acetate, 0.2% acetic acid, 5 μM methylene di-phosphonic acid in water) and mobile phase B [5 mM ammonium acetate, 0.2% acetic acid in 95:5 (v/v) acetonitrile:water] was applied as follows: initial equilibration at 100% B for 1.0 min, linear decrease to 89% B over 10 min, linear decrease to 70% B over 4.75 min, linear decrease to 20% B over 0.5 min, hold at 20% B for 2.25 min, linear increase to 100% B over 0.1 min, re-equilibration at 100% B for 2.4 min. Eluted metabolites were subjected to mass spectrometry analysis on a Q Exactive Hybrid Quadrupole-Orbitrap Mass Spectrometer (Thermo Fisher Scientific) equipped with a HESI-II source probe using Full MS with Data Dependent tandem MS. Source settings were as follows: sheath gas at 55 (arbitrary units), aux gas flow at 20, sweep gas at 2, spray voltage at 3 | kV| spray, capillary temperature at 400C, and S-lens RF at 50. MS1 was set at 70,000 mass resolution, with automatic gain control target at 3.0E06 with a maximum allowed injection time of 100 ms, at a 70–1,050 m/z scan range. dd-MS2 was set at 17,500 mass resolution with automatic gain control target at 1.0E5 and a maximum allowed injection time of 50 ms, a 2 m/z isolation window and stepped normalized collision energies at 10, 20, and 30 (dimensionless units). All data was collected in centroid mode. The scan cycle included a single MS1 scan followed by sequential MS/MS of the top two most intense MS1 ions excluding any fragmented within the previous 10 s. Ions selected for fragmentation must meet a minimum AGC target threshold of 1.0E3 with absolute charge less than four. Each sample was analyzed in negative ionization mode. Sample were injected in randomized order with solvent blank injections between each; internal and external standards were used for quality control purposes and for retention time predictions of compounds from in an in-house standards library. Using custom python scripts and metabolite atlases (Bowen and Northen, 2010; Yao et al., 2015), mass-to-charge ratios, retention times and where possible spectra fragmentation patterns were used to confirm metabolite identification by comparison to metabolite standards analyzed using the same LC-MS/MS methods. Mutant and RCH2 cultures on N1B4 medium were compared with uninoculated control N1B4 spent medium using ANOVA and Tukey HSD in R.

## Results

### Biofilm Interaction Mapping and Analysis Screening: Microbial Interaction Mapping and Strain Selection

As the first step of BIMA, a colony printing method was developed on an automated liquid handling system for the purpose of scalability and transferability between labs. Fourteen preprinted “effector” Pseudomonas strains (including RCH2) were evaluated for their effect on the growth of subsequently printed neighboring colonies of *P. stutzeri* RCH2 (Figures 2A,B). Pseudomonas strains 3–9, and 11 inhibited the growth of the four closest RCH2 colonies, located along the sides of the square of neighboring colonies (Figure 2C). Interestingly, for a subset of these (3, 4, 5), the more distant RCH2 colonies at the corners of the square of neighboring colonies had larger mean areas (Figure 2D). Pseudomonas strain 2 (*Pseudomonas fluorescens* FW300-N1B4) was the only strain that did not inhibit RCH2 (closest side colonies) and enhanced the growth of RCH2 (corner colonies). This strain “N1B4,” was selected for further analysis in co-culture with *P. stutzeri* RCH2.

### Biofilm Interaction Mapping and Analysis Morphology: Co-colony Structure and Infiltration

**FIGURE 3:**
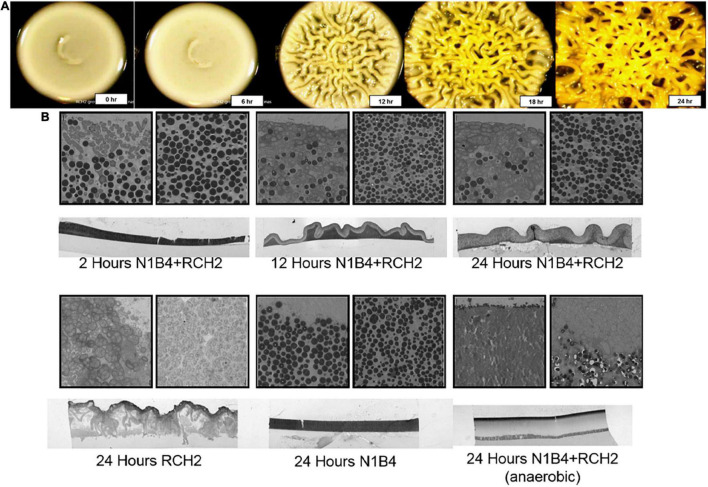
Transmission electron microscopy of co-colony morphology. **(A)** Timepoint images of a co-colony of *Pseudomonas* sp. FW300-N1B4 (underlay) and *Pseudomonas stutzeri* RCH2 (overlay) were taken every 6 h for the first 24 h of the overlay culture. Control N1B4 cultures remain smooth (see Figure 4 and Supplementary Figure 2). **(B)** Transmission electron microscopy imaging (1,400×) of vertical slices showing the co-colony structure, air-colony interface, and strain interface were taken at 2, 12, and 24 h of aerobic RCH2 overlay culture, and at 24 h of an anaerobic RCH2 overlay culture. Similar imaging at 24 h of aerobic RCH2 and N1B4 monocultures were used to determine staining and morphology of each cell type.

Time lapse images of the RCH2 overlay on N1B4 were taken to evaluate the co-colony morphology. Highly wrinkled, rugose colony morphology had formed by 12 h after application of the RCH2 overlay (Figure 3A). The co-colony species interface was further examined using transmission electron microscopy (TEM) (Figure 3B). TEM after 2 h showed a stratification with a clear distinction between RCH2 on the surface and N1B4 underneath. Over the course of 24 h, the colonies became more mixed with infiltration of the N1B4 layer by RCH2. In both the co-culture and in isolate culture, RCH2 biofilm developed sacs containing groups of spherical cells at the air interface; whereas when spotted on the surface of N1B4, single, elongated RCH2 are observed at the N1B4 interface. The sacs became visible in the 24 h co-colony. The anaerobic co-colony culture developed a compact RCH2 layer with no visible EPS sacs.

### Biofilm Interaction Mapping and Analysis Genomics: Mutant Fitness Analysis and Interactions

**FIGURE 4:**
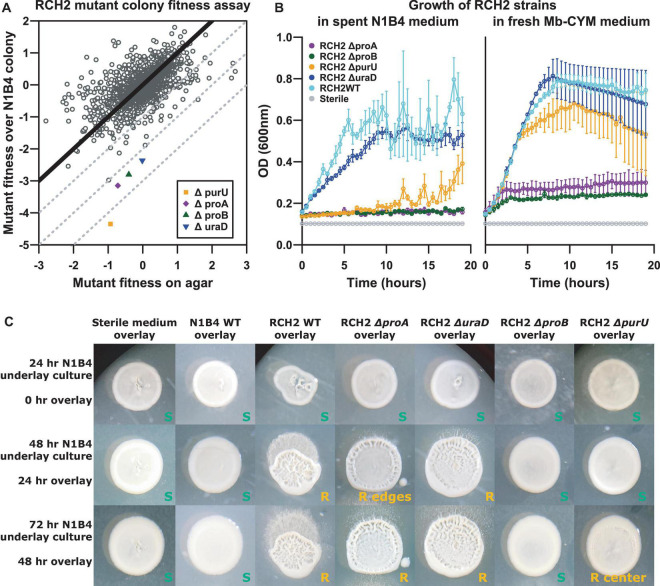
RCH2 mutant co-colony interactions. **(A)** RB-TnSeq mutant library fitness assay, **(B)** mutant growth on N1B4 spent and fresh media, **(C)** mutant growth on N1B4 colonies. All strains of RCH2 grew alone (on control sterile medium underlay) and formed rugose colonies by 24 h of overlay culture (Supplementary Figure 3 and Supplementary Table 5). Images are notated with “S” or “R” for smooth or rugose morphology, respectively; location noted when rugose morphology was located only in the center or periphery of the colony.

A pooled mutant fitness assay was used to identify RCH2 genes essential for successful aerobic co-culture growth with N1B4; the anaerobic culture was not evaluated further since no morphological interaction was observed. The top four genes with the largest differential gene fitness between RCH2 on LB and RCH2 on N1B4, where fitness on N1B4 was negative included gamma-glutamyl phosphate reductase (*proA*), OHCU decarboxylase (*uraD*), formyltetrahydrofolate deformylase (*purU*), and glutamate 5-kinase (*proB*) (Figure 4A). To investigate these genes further, we constructed isogenic single-gene deletion strains for all four genes.

In liquid culture, while all mutants were capable of growth on fresh medium, only the Δ*uraD* deletion strain had similar growth to the wild-type when cultured on spent N1B4 medium (Figure 4B). Minimal to no growth was observed for the Δ*proA* and Δ*proB* deletion mutants, and delayed growth was observed for the Δ*purU* deletion mutant. N1B4 had similar growth on the spent medium of RCH2 wild-type and mutants (not shown). The limited or delayed growth indicated these mutants were unable to acquire some required metabolite from the medium that was consumed by N1B4 in liquid culture or that growth may have been inhibited by something N1B4 was producing.

The biofilm interactions were analyzed by manual printing of the RCH2 wild-type and mutant strains on the top of an established N1B4 colony. The overlays were imaged at 24 and 48 h after overlay printing (Figure 4C). Wild-type RCH2, and Δ*proA* and Δ*uraD* deletion strains formed rugose co-colony biofilms after 24 h similar to the wild-type but with a smoother center for Δ*proA*, while the Δ*purU* mutant had delayed rugose formation and the Δ*proB* mutant remained smooth up to 72 h of observation. All mutants formed rugose colonies after 24 h of overlay on control medium. On its own, the Δ*purD* formed more tubular structures than the wild-type alone, similar to growth on N1B4 for both (Supplementary Figure 3).

### Biofilm Interaction Mapping and Analysis Exometabolomics: Potential for Metabolite Exchange

**FIGURE 5:**
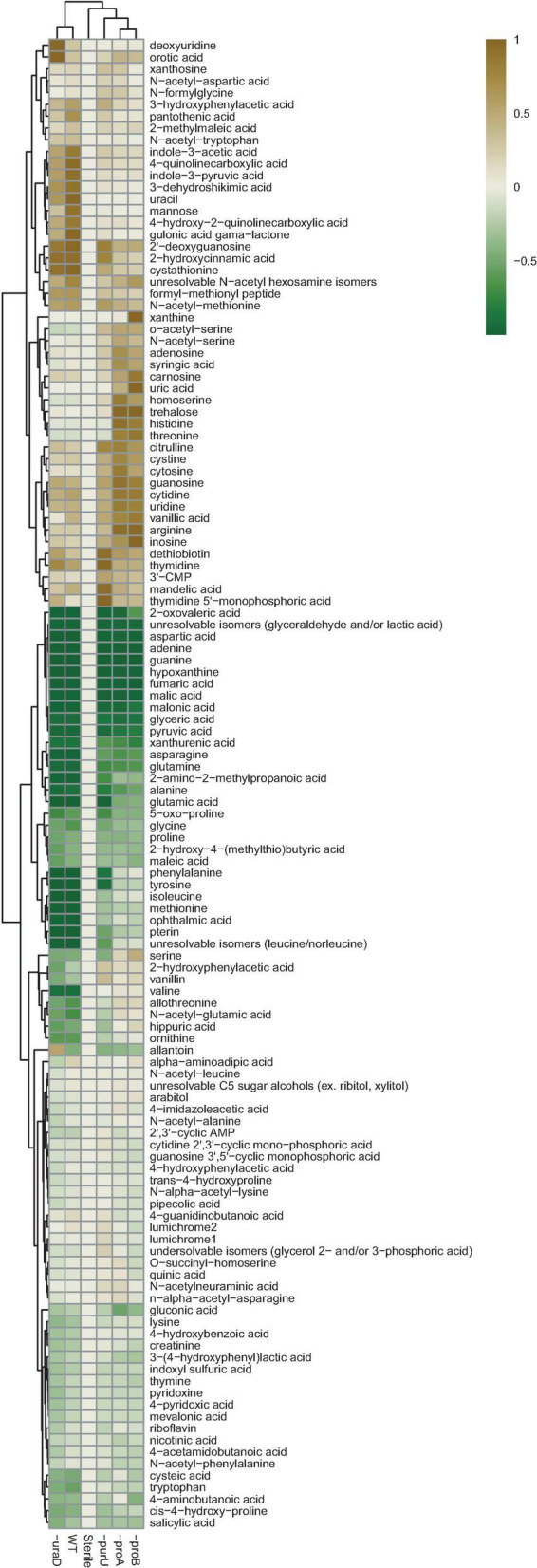
Metabolomics. N1B4 spent medium was sterile filtered and used to culture *Pseudomonas stutzeri* RCH2 wild-type, and mutants Δ*uraD*, Δ*purU*, Δ*proA*, Δ*proB* for 24 h before collection of extracellular metabolites for extraction and LCMS analysis. A media control was incubated alongside the sample cultures to control for contamination and for comparison with starting metabolite abundances. A targeted analysis was performed using a library of m/z, retention time and MS2 spectra of common polar metabolites. Relative peak heights boxplots and statistical comparisons are available in Supplementary Figures 4, 5 and Supplementary Table 6.

To further evaluate why the four RCH2 genes identified in the mutant fitness assay were important to growth in the aerobic co-colony, spent medium from N1B4 was fed to each of the mutants described above and the wild-type RCH2 and then metabolomics was performed to check for altered metabolism in the mutants. Despite limited growth of three of the mutants on N1B4 spent medium (Figure 4B), all four RCH2 mutants appeared to be metabolically active with significant increases and decreases in metabolite abundances relative to uninoculated N1B4 spent medium. In many cases the changes were significantly different than the wild-type (Figure 5 and Supplementary Figures 4, 5). The *P. stutzeri* RCH2 wildtype consumed several N1B4 metabolites involved in one-carbon (C1) metabolism though consumption of methionine was reduced in *P. stutzeri RCH2* strain JWST9066 (Δ*purU*) (Supplementary Figure 5). *P. stutzeri* RCH2 strain JWST9063 (Δ*uraD*) accumulated allantoin, presumably from spontaneous conversion of 2-oxo-4-hydroxy-4-carboxy-5-ureidoimidazoline (OHCU) to (R)-allantoin. *P. stutzeri* RCH2 strain JWST9060 (Δ*proA*) and *P. stutzeri* RCH2 strain JWST9069 (Δ*proB*), had reduced consumption of glutamate (precursor in proline synthesis) from N1B4, when compared to wild-type, and similar consumption of proline (Supplementary Figure 5).

## Discussion

Biofilm formation and morphology in pseudomonas appears to be regulated by a combination of biotic factors including, but not limited to: phenazines, exopolysaccharide production, signaling molecules and flagellar activity (Merritt et al., 2010). In pigmented species of pseudomonas, it has been shown that a reduced cytoplasm (induced under anoxic and low nitrate conditions) as measured by NADH:NAD + ratio results in a rugose or wrinkled colony morphology while under aerobic and normal nitrate conditions, where reduction of phenazines or nitrate to dinitrogen gas occurs, they maintain a smooth morphology (Dietrich et al., 2013). Interestingly, *P. stutzeri*, has a distinguishing morphological characteristic of forming rugose or wrinkled colonies following initial isolation and culture on agar. While brown in color due to cytochrome c, they are classified as a non-pigmented, non-fluorescent pseudomonas and are not known to produce phenazines (Lalucat et al., 2006). *P. stutzeri* can grow anaerobically in the presence of nitrate and has been used for denitrification purposes (Lalucat et al., 2006). However, it has been demonstrated that after repeated culture, smooth colonies can form and it may take several transfers in nitrate media under semi-aerobic conditions before they are able to grow under denitrifying anaerobic conditions (Lalucat et al., 2006).

Using BIMA, we were able to screen colony forming microbial effector strains for growth promoting and inhibiting effects on *P. stutzeri.* TEM imaging revealed unique cellular morphologies of the *P. stutzeri* RCH2 on the surface of *P. fluorescens* FW300-N1B4. In other *P. stutzeri* strains, exopolysaccharide sacs have been shown to be associated with oxygen exclusion for nitrogen fixation under aerobic conditions (Wang et al., 2017). However, we observed a loss of sac formation at the species interface, suggesting possible exchange of nitrogen containing compounds from N1B4 to RCH2 obviating the need for nitrogen fixation. We observed that N1B4 metabolites supported the growth of RCH2 and metabolomics analysis confirmed consumption of its metabolites. The mutant analysis provided further support for this view. Specifically, we saw that the Δ*purU* mutant’s consumption of metabolites was altered and its growth was inhibited. Given that PurU is important in maintenance of one carbon pools, the Δ*purU* mutant may be unable to obtain the reaction products (THF and formate) from N1B4, and as suggested by previous studies in *E. coli*, may experience glycine starvation due to GlyA inhibition by methionine and adenine (Nagy et al., 1995), both metabolites RCH2 took up from N1B4 based media. In the Δ*uraD* mutant, which accumulated allantoin, presumably (R)-allantoin, RCH2 would be unable to use this in downstream nitrogen assimilation pathways without a racemase (Bovigny et al., 2014), an enzyme *P. stutzeri* has been shown to lack (Van Der Drift et al., 1975). Thus UraD is probably essential for converting OHCU into usable (S)-allantoin. In some bacterial species, the ureide pathway is utilized for recovery of nitrogen from purines under stress conditions (Serventi et al., 2010; Izaguirre-Mayoral et al., 2018). Under aerobic conditions or presence of ammonia, N2 fixation in *P. stutzeri* is suppressed and nitrification is active, however, in biofilm, nitrogen fixation is suspected to occur in the EPS sacs produced by the rugose morphology (Yan et al., 2010; Wang et al., 2017). At the N1B4—RCH2 interface of the co-colony (which lacked EPS sacs in imaging) and in liquid culture with shaking and sufficient gas exchange, nitrogen fixation may be suppressed in favor of utilization of exchanged organic nitrogen compounds such as intermediate allantoin precursors (5-hydroxyisouric acid or OHCU) from N1B4 spent medium, prior to conversion to usable (S)-allantoin. With the Δ*proA and* the Δ*proB* mutants, both known auxotrophs for proline,^1^ we observed that while proline was still consumed, glutamate was not to the same extent as wildtype. The wild-type may use exchanged glutamate for proline synthesis while the proline synthesis mutants are likely unable to obtain enough proline directly from N1B4 and is likely unable to sufficiently utilize alternative sources such peptide degradation or by alternative synthesis from ornithine (Supplementary Figure 5). Together, these results indicate the growth of the RCH2 may be reliant upon uptake of metabolites produced by the N1B4.

While our experiments focused on cooperative interactions, inhibitory interactions may be of interest in controlling pathogenic strains in agricultural systems. The inhibitory interactions of certain pseudomonas species have been studied in detail. For example, some pathogenic species of pseudomonads produce lipodepsipeptides (e.g., corpeptins from *P. corrugata*, a plant pathogen) which also have antimicrobial activity (Raaijmakers et al., 2010). These may contribute to interspecific competition between pseudomonads by inhibiting biofilm formation and breaking down existing biofilm (Kuiper et al., 2004). An interesting follow up study involving a mutant library of *Pseudomonas corrugate N2F2*, may elucidate the mechanism of inhibition between the two species and the nature of its competitive and pathogenic behavior in nature. Additionally, some beneficial strains such as *P. aureofaciens*, an anti-fungal symbiont of wheat, releases phenazines in the rhizosphere which inhibit the growth of plant pathogens in response to exogenously diffusible signaling molecules (Pierson and Pierson, 1996). These compounds may contribute to competitiveness and the ability to form biofilms (Ramos et al., 2010). Interestingly, phenazines may have multiple functional roles (Mavrodi et al., 2006), including involvement of the redox state of the cytoplasm (Dietrich et al., 2013; Glasser et al., 2014); interactions such as these may be important determinants of co-colony biofilm structure under varying oxygenic conditions.

In this proof of concept study, we focused on existing isolates in co-culture studied under ideal lab growth conditions. We recognize the importance of extending this method in future work to more diverse isolates representing the field microbiome. However, community studies present additional challenges in determining metabolic functions associated with specific microbes. Further, in using BIMA to uncover more environmentally relevant metabolic activities, growth conditions should represent environmental parameters as closely as possible (for example, the use of soil extract, soil based synthetic medium or sterilized soil for microbial growth) (Jenkins et al., 2017; Swenson et al., 2018). The development of ecologically relevant media based on analysis of soil and sediment metabolites is an important future direction for increasing the ecological relevance of these microbial interaction studies. Analytical methods for metabolite extraction and detection should be selected to ensure proper coverage of metabolites of interest (Mushtaq et al., 2014; Louie et al., 2020).

In this work we have demonstrated that bacterial printing can be used to rapidly screen for macroscopic interactions such as changes to colony morphology, size and color. We take advantage of a mutant fitness library and metabolomics to gain insights into genes and compounds mediating observed bacterial interactions. We anticipate that this approach integrating bacterial printing, mutant fitness libraries, targeted genetics, and metabolomics is suitable to investigating diverse microbial interactions. In some instances, cooperative growth may be favorable (such as for bioremediation purposes where both consortia and biofilm-based systems are used). In other cases, competitive interactions may be desired for biocontrol-based purposes or for understanding competitive interactions in soil environments. Knowledge of the genomic and metabolic determinants involved in these interactions allows for more directed design of co-colony-based systems and a better understanding of those existing in nature. We foresee BIMA becoming a valuable tool for the enhancement and understanding of *P. stutzeri* and other co-colony-based systems.

## Data Availability Statement

Raw LCMS data are available from the JGI Genome Portal^2^ under project number 1278333.

## Author Contributions

SK, PR, and TN wrote the manuscript. SK, PR, KW, MZ, GZ, MA, and JZ performed the experiments. SK and BB performed data analysis. MR made revisions. RC maintained and provided bacterial strains. JW, AA, AD, and TN oversaw study and made critical revisions. All authors reviewed and approved the manuscript.

## Conflict of Interest

MR and MZ, currently employed at DiCe Molecules and Molecular Devices, respectively, were not employed there at the time of the study. The remaining authors declare that the research was conducted in the absence of any commercial or financial relationships that could be construed as a potential conflict of interest.

## Publisher’s Note

All claims expressed in this article are solely those of the authors and do not necessarily represent those of their affiliated organizations, or those of the publisher, the editors and the reviewers. Any product that may be evaluated in this article, or claim that may be made by its manufacturer, is not guaranteed or endorsed by the publisher.

## Footnotes

1 http://fit.genomics.lbl.gov/ (accessed 05/14/2020).

2
genome.jgi.doe.gov
